# Various Neurological Symptoms Associated with Infected Internal Iliac Artery Aneurysm: A Case Report

**DOI:** 10.3400/avd.cr.21-00013

**Published:** 2021-06-25

**Authors:** Kaoru Uchida, Takashi Shuto, Tomoyuki Wada, Hideyuki Tanaka, Shinji Miyamoto

**Affiliations:** 1 Department of Cardiovascular Surgery, Faculty of Medicine, Oita University, Yufu, Oita, Japan

**Keywords:** infected aneurysm, neurological symptom, aneurysm ruptured

## Abstract

A 68-year-old man presented with a chief complaint of left leg pain; he was later diagnosed with an infected left internal iliac artery aneurysm. Multiple mononeuropathy was suspected. Since the aneurysm had a high risk of rupture, emergency Y-graft replacement was performed. *Bacteroides vulgatus* was then detected from the pus of the aneurysm. With continuous oral antimicrobial agents following intravenous antimicrobial agents, the patient was noted to have no recurrence. However, his leg pain symptoms continued postoperatively; thus, a supporting device was needed. It should be noted that even neurological symptoms may indicate the presence of aortoiliac aneurysms.

## Introduction

Although internal iliac artery aneurysms are often asymptomatic, symptoms such as abdominal pain; hip, buttock, and inguinal pain; urinary tract compression symptoms; lower limb pain, abdominal symptoms due to compression; enteric fistula of the intestinal tract; melena; and peritonitis have been reported.^1)^ In this study, we report, with literary considerations, on our experience regarding a rare infected left internal iliac aneurysm exhibiting various neurological symptoms.

## Case Report

**Figure 1:**
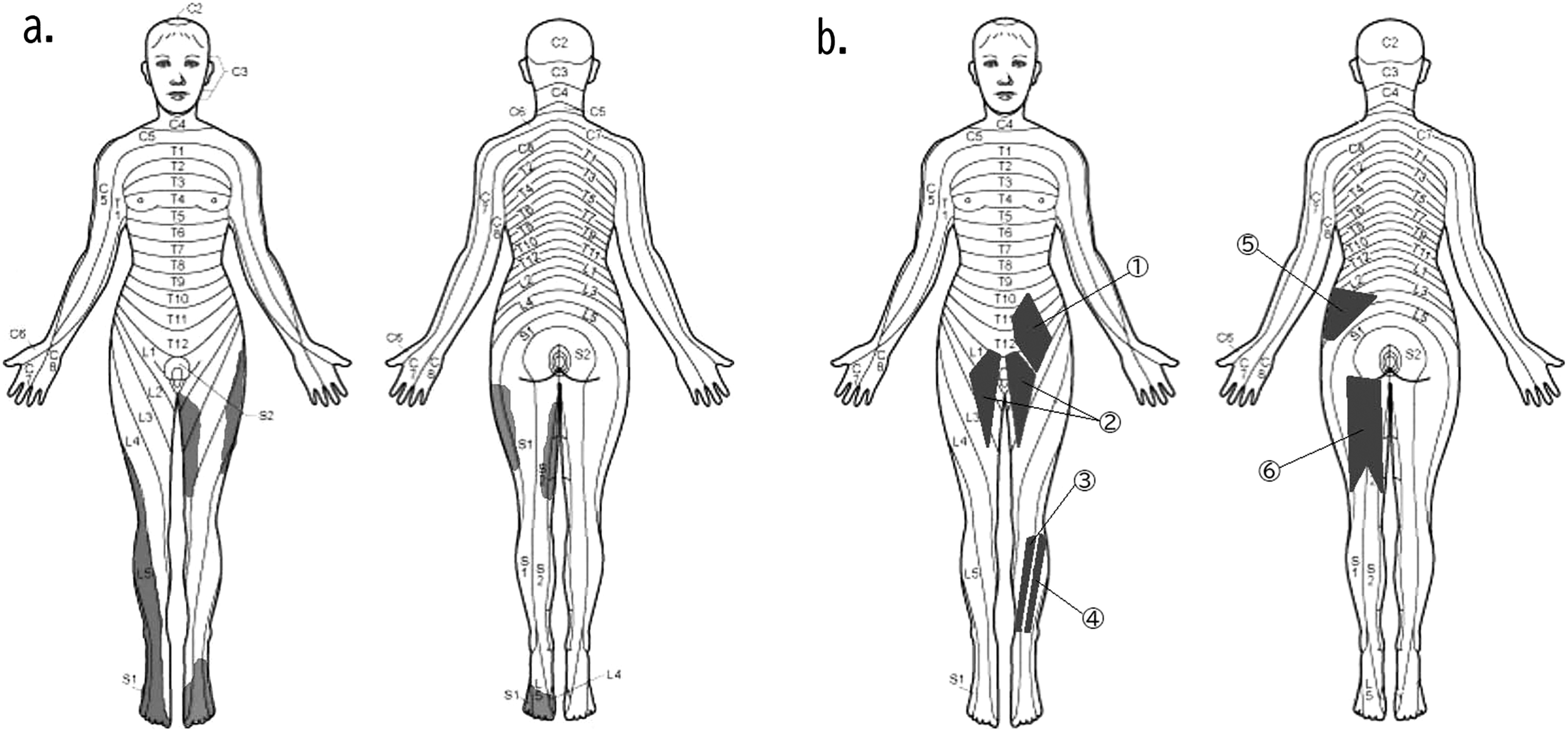
Fig. 1 (**a**) Sensory impairment regions. (**b**) Weakened muscles.

**Figure 2:**
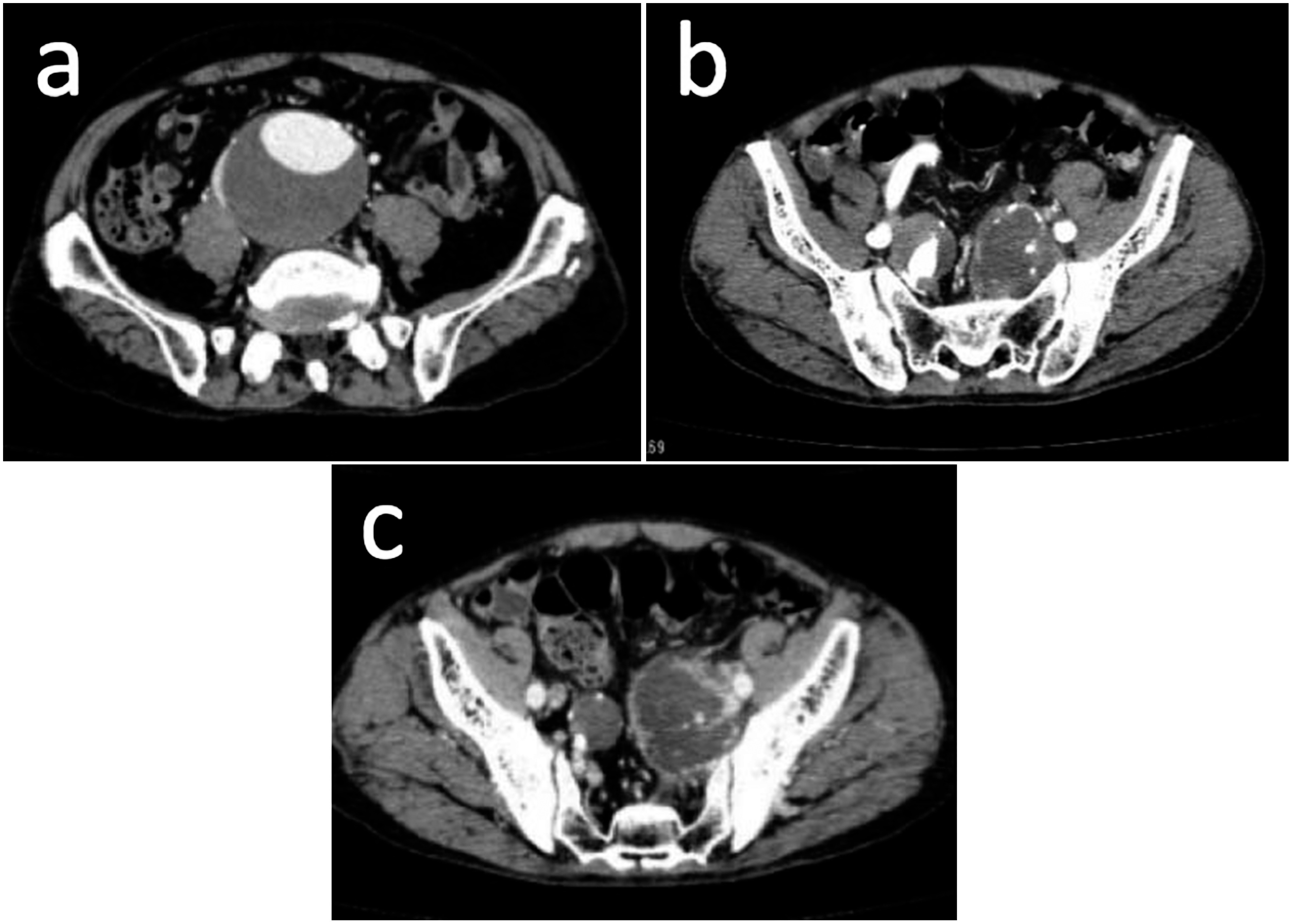
Fig. 2 (**a**) A spindle-shaped abdominal aortic aneurysm with a diameter of 68 mm. (**b**) A right internal iliac aneurysm in contact with the pelvis. (**c**) A left internal iliac artery aneurysm exhibiting marginal irregularities and a lobular shape.

**Figure 3:**
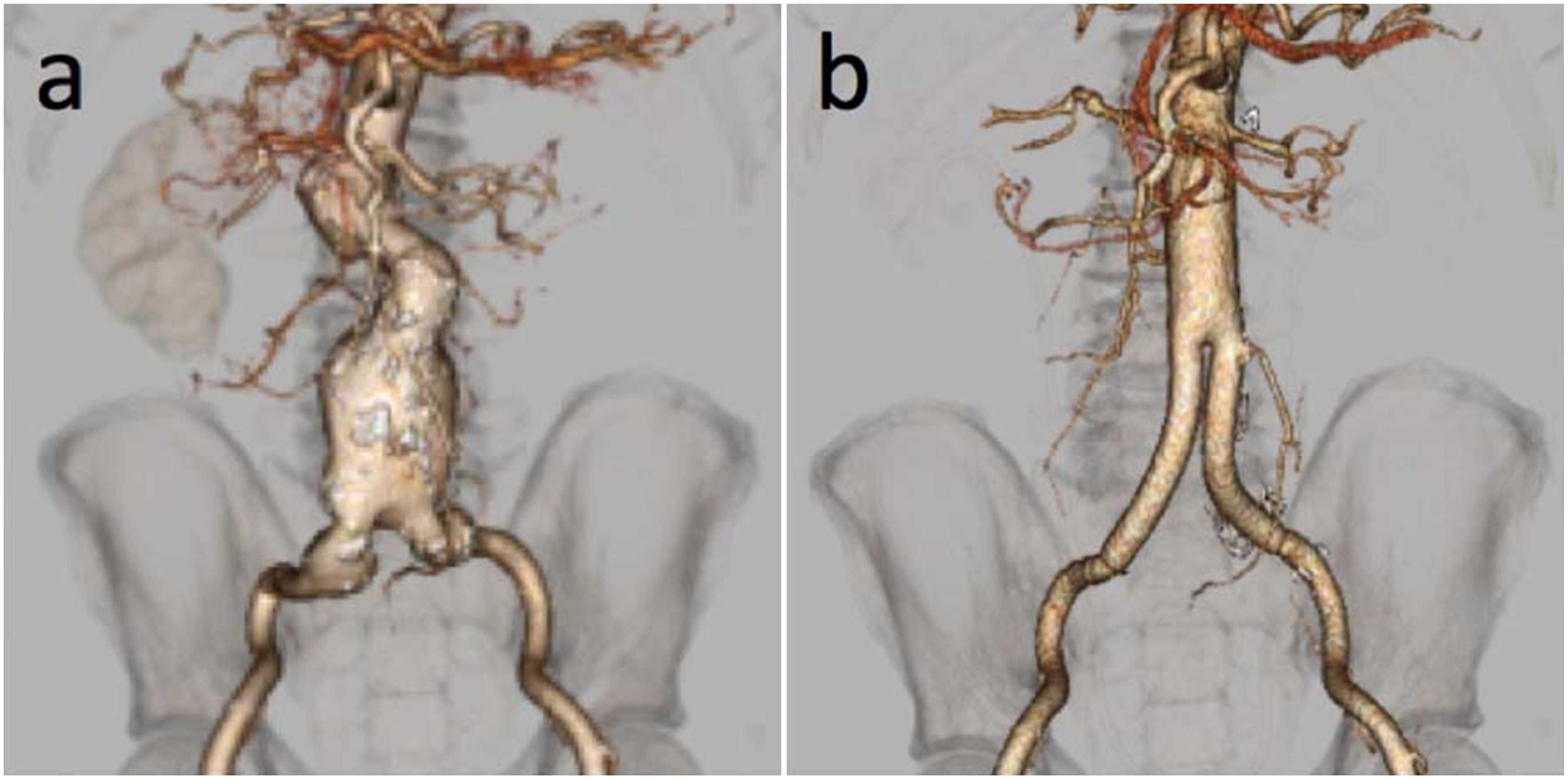
Fig. 3 (**a**) Preoperative 3-dimensional computed tomography (3DCT). (**b**) Postoperative 3DCT.

A 68-year-old man was referred by a practicing orthopedic surgeon to our department with severe left ankle joint pain; as per his magnetic resonance imaging findings, which was undertaken for a lumbar vertebral examination, a large abdominal aortic aneurysm was detected. He had been complaining of left ankle and hip joint pain for the past 2 months. As a high inflammatory response (C-reactive protein 19.32 mg/dl, white blood cell 18110/µl, and a fever) strongly indicates an infected aneurysm, he was admitted to our hospital immediately. There was nothing special in his medical history. He was determined to be a heavy alcohol drinker but had no history of smoking. A pulsatile tumor was felt on his abdomen. He could not walk because of left lower leg weakness, especially in the ankle joint, which had virtually no dorsiflexion. He experienced pain in the left ankle joint although it showed no redness, swelling, or heat. He has poor oral health and had many caries. A neurologist found sensory disorders below the right lower limb, the left thigh medial lateral, and the left ankle joint (**Fig. 1a**). This indicated disorder of the right L5-S1 root, the left lateral femoral nerve, the left femoral nerve anterior cutaneous branch, and the left medial plantar nerve. A manual muscle strength test conducted by a neurologist revealed that the iliopsoas muscle was 5/5, the adductor muscle 4/2, the abductor muscle 5/2, the gluteus maximus muscle 5/3, the quadriceps femoris muscle 5/5, the hamstring muscle 5/4, the anterior tibial muscle 5/0, the toe extensor muscle 5/0, and the gastrocnemius muscle 5/5 (**Fig. 1b**). Muscle weakness indicated disorder of the shallow peroneal nerve, the deep peroneal nerve, and the tibial nerve. Blood biochemical test showed mild hepato-renal dysfunction (aspartate transaminase 61.3 U/l, alanine transaminase 52.2 U/l, blood urea nitrogen 26.8 mg/dl, creatinine 1.48 mg/dl). Endocarditis was negative on echocardiography. No pathogens were detected in the preoperative blood culture. Contrast computed tomography revealed a spindle-shaped aneurysm with a diameter of 68 mm from just below the renal artery to the bilateral common iliac arteries. The bilateral internal iliac arteries were also aneurysmal (right 35 mm, left 52 mm). Both the right and left internal iliac aneurysms were determined to be in contact with the pelvis. The left internal iliac aneurysm displayed marginal irregularities and lobulation. The left ureter was dilated on the central side of the aneurysm, and left hydronephrosis was found (**Figs. 2** and **3a**).

① the gluteus maximus muscle, ② the adductor muscle, ③ the anterior tibial muscle, ④ the toe extensor muscle, ⑤ the abductor muscle, ⑥ the hamstring muscle.

Administration of doripenem (doripenem, 0.5 g×2/day) was initiated from the first day of hospitalization. Since the aneurysms, especially the left iliac artery aneurysm, had a high risk of rupture, we performed graft replacement on the second day after admission.

Surgical findings: A ureteral stent was placed for left hydronephrosis prior to the start of the surgery. The left internal iliac artery aneurysm was covered with inflammatory tissue, which was compressing the left ureter. The pus underneath the inflammatory tissue was then submitted to a culture test. Both the internal iliac aneurysms were incised, while the wall was excised as much as possible. The left internal aneurysm was partially missing the aortic wall, and the branches of the left internal iliac artery had been occluded. A bifurcated graft (GelsoftPlus Bifurcate, TERUMO, Tokyo, Japan) was implanted with peripheral anastomosis performed on both the left and right external iliac arteries. The internal iliac artery was difficult to reconstruct on both the left and right, so the peripheral branches were just closed using sutures (**Fig. 3b**). The inferior mesenteric artery was reconstructed. The graft was entirely covered with the omentum.

*Bacteroides vulgatus* was detected from the culture of the pus, so the administration of DRPM (0.5 g×2/day) was continued. Consequently, the inflammatory response declined steadily; on the 43rd postoperative day, the drug was switched to the oral administration of ampicillin/sulbactam (375 mg×3/day)+clindamycin (150 mg×3/day). The double J catheter was withdrawn on the 37th day following the operation. There were no signs of infection relapse, and the patient was discharged from the hospital on the 52nd postoperative day. His pain and the decrease in motor sensation of the left foot remained as sequelae following the operation, whereas the neurological symptoms of the right leg disappeared at discharge. The pain continued to require the oral administration of pregabalin 350 mg/day. However, orthosis allowed him to be ambulatory.

## Discussion

There have been few reports on neuropathy related to the internal iliac aneurysm. Nachbur et al. reported that 7 (13.2%) of 53 patients with isolated iliac aneurysms presented with neuralgia caused by compression of the sciatic or femoral nerve.^2)^ In other reports regarding non-infected iliac aneurysm, the chief complaint of lower limb pain or abnormal sensation without movement disorders was thought to be sciatica, which was provoked by compression due to aneurysm. Although one patient died of aneurysm rupture,^3)^ the other patients underwent surgery, and the symptoms disappeared thereafter.^4)^ CT findings of internal iliac aneurysm presenting with neuropathy include an aneurysm large enough to get in contact with the pelvis,^4)^ and a saccular pseudoaneurysm secondary to Behcet’s disease, kidney transplantation, and trauma^5–9)^ rather than a true aneurysm.

In our case, the patient chiefly complained of ankle pain and difficulty in dorsiflexion in the left leg. Muscle weakness was mainly found in the left leg, but sensory impairment was confirmed in both legs. On the right leg, the sensory impairment was consistent with the areas of the L5 to S1 nerve root. These nerve roots could be simply compressed by the right internal iliac aneurysm. The sensory impairment was also associated with radiculopathy and disappeared after the removal of the aneurysm. The clinical course of the right leg was in line with that in the abovementioned previous reports.

In contrast, the areas of sensory impairment of the left leg were consistent with the part of the peripheral nerves including the femoral and the medial plantar nerve, which did not coincide with the areas of the nerve root. The muscle weakness in the left leg also seemed to be the impairment of the peripheral nerves such as the shallow peroneal nerve, the deep peroneal nerve, and the tibial nerve. These sensory and motor impairments indicated peripheral neuropathy of L4 to S1. The peripheral neuropathy of the left leg has remained even after the operation unlike on the right. There have been no reports in which irreversible neuropathy is caused by infected aneurysms as shown here.

Considering both the symptoms on the left leg unrelated to the simple radiculopathy and the pathology of infection, multiple mononeuropathy by infection-related vasculitis is the most likely suspect. The pathophysiology of vasculitis causing mononeuropathy is multiple infarctions of small-arteriolar occlusion, which, in turn, nourishes the peripheral nerves.^10)^ Multiple mononeuropathy, therefore, leads to irreversible neurological symptoms as shown in our case. If the neurological infarction is caused by systemic thrombus related to sepsis, multiple mononeuropathy should be seen systematically. In our case, the mononeuropathy was localized in the left leg, demonstrating that the neurological infarction was most likely caused by local vasculitis associated with infected aneurysm.

## Conclusion

An infected left internal iliac artery aneurysm has been determined to result in various neurological symptoms that could not be explained by radiculopathy alone. Multiple mononeuropathy due to spreading inflammation to neurotrophic vessels might be its cause. It should be noted that intricate neurological symptoms of lower limbs may indicate the presence of aortoiliac aneurysms involving infection.

## Abbreviations

CRP: C-reactive protein
WBC: White blood cell
CT: Computed Tomography
